# Safety of the BNT162b2 mRNA COVID-19 vaccine in children and adolescents with juvenile idiopathic arthritis: a tertiary-center early experience

**DOI:** 10.1186/s43166-022-00143-z

**Published:** 2022-07-18

**Authors:** Abobakr A. Abdelgalil, Reima A. Bakry, Mohammed A. Muzaffer

**Affiliations:** 1 Department of Pediatrics, King Abdulaziz University, Jeddah, Saudi Arabia; 2 Department of Pediatrics, Cairo University, Cairo, Egypt; 3 Department of Pediatrics, East Jeddah Hospital, Jeddah, Saudi Arabia

**Keywords:** Juvenile idiopathic arthritis, SARS-CoV-2, mRNA COVID-19 vaccine, Safety, Children

## Abstract

**Background:**

Research on the COVID-19 vaccination in patients with underlying rheumatic disorders in pediatric age is lacking. We studied possible adverse events of the mRNA BNT162b2 vaccine against SARS-CoV-2 (Pfizer-BioNTech) in children and adolescents with juvenile idiopathic arthritis (JIA), and also if there is a risk of flaring of the underlying JIA. We reported 36 JIA patients aged 5–18 years old received 2 doses of the COVID-19 vaccine (72 doses). Patients were followed before and after vaccination, and any related adverse event was recorded. JIA disease activity was assessed using Juvenile Arthritis Disease Activity Score-10 (JADAS-10) before and after vaccination.

**Results:**

Among 72 doses of the vaccine received, local adverse events (AEs) were reported by majority of the patients (66.7%); most commonly reported local AE was pain at the site of injection. Systemic AEs were revealed by (65.3%), most commonly reported systemic AEs were tiredness, myalgia, and headache. Almost all the reported AE were mild to moderate and resolved within 1–2 days and were also more frequently noted after the second dose. No flaring of the underlying primary rheumatic disease after vaccination. No one of the study group revealed serious adverse events.

**Conclusions:**

This is one of the early studies reporting that mRNA COVID-19 vaccine seems to be safe in children and adolescents with JIA. Almost all the reported adverse events were mild to moderate and transient. Also, no serious adverse events or flaring of the primary disease were reported.

## Background

COVID-19 pandemic is paralyzing the entire world causing significant illness and death [1]. Worldwide, as of 14 April 2022, there have been more than 500 million SARS-CoV-2 infections, including more than 6 million deaths [2]. Although SARS-CoV-2 infections are usually mild among children and adolescents, severe disease can occur with possible hospitalization, intensive care unit (ICU) admission and around 4% of those hospitalized require mechanical ventilation. Moreover, despite the fact that SARS-CoV-2 related multisystem inflammatory syndrome in children (MIS-C) is uncommon; it is serious and can be potentially fatal in the pediatric age group [3]. More importantly, children with underlying chronic conditions, particularly those with autoimmune and auto-inflammatory illnesses like juvenile idiopathic arthritis (JIA), may experience a similar or slightly higher risk for COVID-19 disease than the general population. They may be, however, at a higher risk of a more severe disease course, including prolonged viremia, autoantibody production, and death [4].

So far, the absence of specific treatment for this potentially fatal COVID-19 infection makes prevention strategy the gold standard, in particular for this vulnerable age group [5]. The Food and Drug Administration (FDA) has recently extended use authorization of the messenger RNA (mRNA) vaccine BNT162b2 (Pfizer-BioNTech COVID-19 vaccine) to adolescents aged 12 and above on May 2021 and then to children 5 through 11 years of age on October 2021 [6]. Although there is a theoretical risk of autoimmunity flare in patients with rheumatic diseases and risk of improper vaccine induced antibody response, vaccination data from adult populations with autoimmune diseases are promising, with minor adverse events, no significant flare, and furthermore a positive antibody response in patients on non B cell depleting anti- rheumatic drugs [7]. Although the vaccines in general are ideally recommended at times of non flares of autoimmune diseases, some authors recommend receiving this non-lived mRNA vaccine against SARS-CoV-2 regardless disease activity (except for life threatening conditions) and even additional third vaccine dose may be planned 4 weeks after completing the principal two vaccine doses [8].

Research on the COVID-19 vaccination of patients with underlying rheumatic disorders has been mostly restricted to adults and studies concerning pediatric age are lacking. Patients and physicians are preoccupied with inquiries, particularly concerning the vaccination’s safety in pediatric patients with rheumatic disorders. We aimed to detect safety and possible adverse events of this mRNA vaccine against SARS-CoV-2 (Pfizer-BioNTech) in children and adolescents with JIA after receiving the 2 main doses of the vaccine and also if there is a risk of flaring of the underlying JIA trying to settle the decision of the vaccination.

## Methods

This cross-sectional prospective study was conducted at a tertiary hospital. Patients aged 5–18 years old known as JIA following in pediatric rheumatology clinic represented the target group of the study. A convenience sampling was used to include all eligible patients (36 patients) who received two consecutive doses of COVID-19 vaccine among 42 patients who presented for follow up from August 8th 2021 till February 6th 2022. Patients who had a high Juvenile Arthritis Disease Activity Score-10 (JADAS-10) disease activity were excluded. Also those who refused vaccination or did not complete the two vaccine doses were excluded. The participants received two doses of the COVID-19 vaccine (Pfizer-BioNTech) intramuscularly 3 weeks apart. All patients were observed 30 min after receiving each dose, for possibility of immediate adverse reaction, and an electronic diary was sent to them to record any early local or systemic adverse event occurring during the 14 days following each vaccine dose (including yes/no questions). Also, they were informed to report serious adverse events that necessitated hospitalization for 2 months after vaccination. Also, they reported if they had COVID-19 infection at any time whether before or even after the vaccination.

Demographic data, clinical data, disease course, and current medications were obtained from our hospital computer system. The study patients were followed and disease activity was assessed using JADAS-10 score within one month before and also within 1 month after the vaccination. This score was used to detect effect of the vaccine in flaring JIA manifestations. The JADAS-10 is calculated by examining four parameters (1) the attending physician’s overall assessment of disease activity (using a visual analog scale (VAS) where 0 means no activity and 10 means maximum activity); (2) parent/child assessment of well-being (using a VAS; where 0 means very well and 10 means very poor); (3) active joints number (0–10), and (4) ESR (0–10) (ESR (mm/h) − 20)/10), for a total score of 0 − 40. Inactive, low disease activity, moderate disease activity, and high disease activity were divided into four categories based on cutoff values of 1, 1.1–3.8, 3.9–10.5, and > 10.5 correspondingly [9]. All current medications were continued apart from methotrexate which was withheld only 1 week after each vaccine dose as well as NSAIDs which were withheld 24 h before the vaccine dose according to recommendations of American College of Rheumatology (ACR) [8].

### Ethical consideration

Our Institutional Review Board’s Research Ethics Committee approved it (Reference. number 342–21). The study was carried out in line with the Helsinki Declaration. Completion of the electronic diary was considered as a consent for participation in the study.

### Statistical analysis

The statistical package for the social sciences (SPSS) version 26 was used to code and enter the data (IBM, Armonk, New York). Quantitative data were represented as mean, standard deviation together with median, minimum, and maximum and compared using the Mann–Whitney test. Nominal data were summarized as frequency and percentage and compared utilizing chi-square test. The non-parametric Wilcoxon signed-rank test was used to compare serial measurements within each patient; and if frequency is suspected < 5, the exact test was utilized instead. Statistical significance was set as a *P* value < 0.05.

## Results

A total of 36 JIA patients 5 to 18 years of age were enrolled. The mean (SD) age was 13.3 ± 3.1 years. Females compromised 66.7% of the cohort. The median disease duration was 4.5 (IQR 0.3–11) years. Almost all the patients were stable; either in remission, low or moderate disease activity before vaccination; median JADAS-10 was one (IQR 0.0–4.5). The most used DMARD was methotrexate (41.7%). Only three patients (8.3%) reported SARS-CoV-2 infection few weeks after the second dose of the vaccine, confirmed by PCR and it was mild infection. Baseline demographic and clinical data of the participants were shown in Table 1.

**Table 1:** JIA patients’ baseline characteristics

Characteristics	*N* = 36 patients	95% CI
Age (years), mean (SD)	13.3 (3.1)	
Females	24 (66.7%)	50.5–80.3
**JIA subtype**		
Oligo-articular JIA	12 (33.3%)	19.7–49.5
Poly-articular JIA	10 (27.8%)	15.3–43.7
Systemic JIA	8 (22.2%)	11.1–37.6
ERA	5 (13.9%)	5.5–27.8
Psoriatic arthritis	1(2.8%)	0.3–12.3
Disease duration (years), median (range)	4.5 (0.3–11.0)	
**Medications**		
NSAIDs	5 (13.9%)	5.5–27.8
Methotrexate	15 (41.7%)	26.7–57.9
Cyclosporine	2 (5.6%)	1.2–16.6
Adalimumab	11 (30.6%)	17.4–46.7
Etanercept	7 (19.4%)	9.1–34.4
Anakinra	1 (2.8%)	0.3–12.3
Tocilizumab	4 (11.1%)	3.9–24.3
Infliximab	1 (2.8%)	0.3–12.3
Off medications	7 (19.4%)	9.1–34.4
**Associated comorbidity**	8 (22.2%)	11.1–37.6
Obesity	1 (2.8%)	0.3–12.3
IDDM	1 (2.8%)	0.3–12.3
Hypothyroidism	1 (2.8%)	0.3–12.3
ADHD	1 (2.8%)	0.3–12.3
Short stature	1 (2.8%)	0.3–12.3
IBD	1 (2.8%)	0.3–12.3
Hyperlipidemia	1 (2.8%)	0.3–12.3
HIV	1 (2.8%)	0.3–12.3
Previous COVID-19 infection	5 (13.9%)	5.5–27.8
COVID-19 infection after vaccination	3 (8.3%)	2.4–20.6

*Abbreviations: ADHD* Attention deficit hyperactivity disorder, *COVID-19* Coronavirus disease 2019, *ERA* Enthesitis-related arthritis, *HIV* Human immunodeficiency virus, *IBD* Inflammatory bowel disease, *IDDM* Insulin-dependent diabetes mellitus, *JADAS-10* Juvenile Arthritis Disease Activity Score-10, *JIA* Juvenile idiopathic arthritis, *NSAIDs* Non-steroidal anti-inflammatory drugs, *SD* Standard deviation

Among 72 doses of the BNT162b2 vaccine received, almost all the reported local (66.7%) and systemic (65.3%) adverse events (even at least one AE) were mild to moderate and resolved within 1–2 days. Pain at site of injection was the most common local AE (62.5%), while tiredness was the most frequent systemic one (48.6%), followed by myalgia and headache (44.4% and 41.7%) respectively. Painful axillary lymphadenopathy was reported only in one patient (1.4%) after receiving the second dose. No one of the cohort exhibited serious AE that necessitated hospitalization. Four patients (five doses (6.9%)) developed hives following the vaccination; one patient after both doses while the other three patients after the second dose only, relieved by just anti-histamines and local creams. Similarly, four patients (five doses (6.9%)); developed mild chest pain; relieved by NSAIDs only or self-limited, and one of them developed mild to moderate chest pain after both doses relieved by 2 days NSAIDs and oral steroids prescribed by the primary physician via teleconsultation. Post-vaccination AE were shown in Table 2.

**Table 2:** Post-vaccination adverse events (AE)

**Post-vaccination adverse events (AEs)**	**AE after first dose *n* = 36(%)**	**AE after second dose *n* = 36**(%)	**Total *n* = 72 doses (%)**	**95% CI**
**Local AEs (at least one AE)**	24 (66.7%)	24 (66.7%)	48 (66.7%)	55.3–76.7
Pain at site of injection	22 (61.1%)	23 (63.9%)	45 (62.5%)	51.0–73.0
Swelling	8 (22.2%)	9 (25.0%)	17 (23.6%)	15.0–34.3
Erythema	9 (25.0%)	8 (22.2%)	17 (23.6%)	15.0–34.3
Ipsilateral painful LNs	0 (0.0%)	1 (2.8%)	1 (1.4%)	0.2–6.3
**Systemic AEs (at least one AE)**	22 (61.1%)	25 (69.4%)	47 (65.3%)	53.9–75.5
Fever	9 (25.0%)	16 (44.4%)	25 (34.7%)	24.5–46.1
Chills	4 (11.1%)	9 (25.0%)	13 (18.1%)	10.5–28.1
Headache	16 (44.4%)	14 (38.9%)	30 (41.7%)	30.8–53.2
Diarrhea	0 (0.0%)	0 (0.0%)	0 (0%)	0–5
Nausea	7 (19.4%)	7 (19.4%)	14 (19.4%)	11.6–29.7
Myalgia	15 (41.7%)	17 (47.2%)	32(44.4%)	33.4–56.0
Tiredness	15 (41.7%)	20 (55.6%)	35 (48.6%)	37.3–60.0
New transient joint pain	3 (8.3%)	6 (16.7%)	9 (12.5%)	6.4–21.6
Allergy	1 (2.8%)	4 (11.1%)	5 (6.9%)	2.7–14.6
Subjective flaring of 1ry disease	1 (2.8%)	0 (0.0%)	1 (1.4%)	0.2–6.3
Chest pain	2 (5.6%)	3 (8.3%)	5 (6.9%)	2.7–14.6
Serious AE	0 (0.0%)	0(0.0%)	0 (0%)	0–5
Adverse events	29 (80.6%)	31 (86.1%)	83.3	73.5–90.6

*Abbreviations: AE* Adverse event, *LN* Lymphadenopathy

**Fig. 1:**
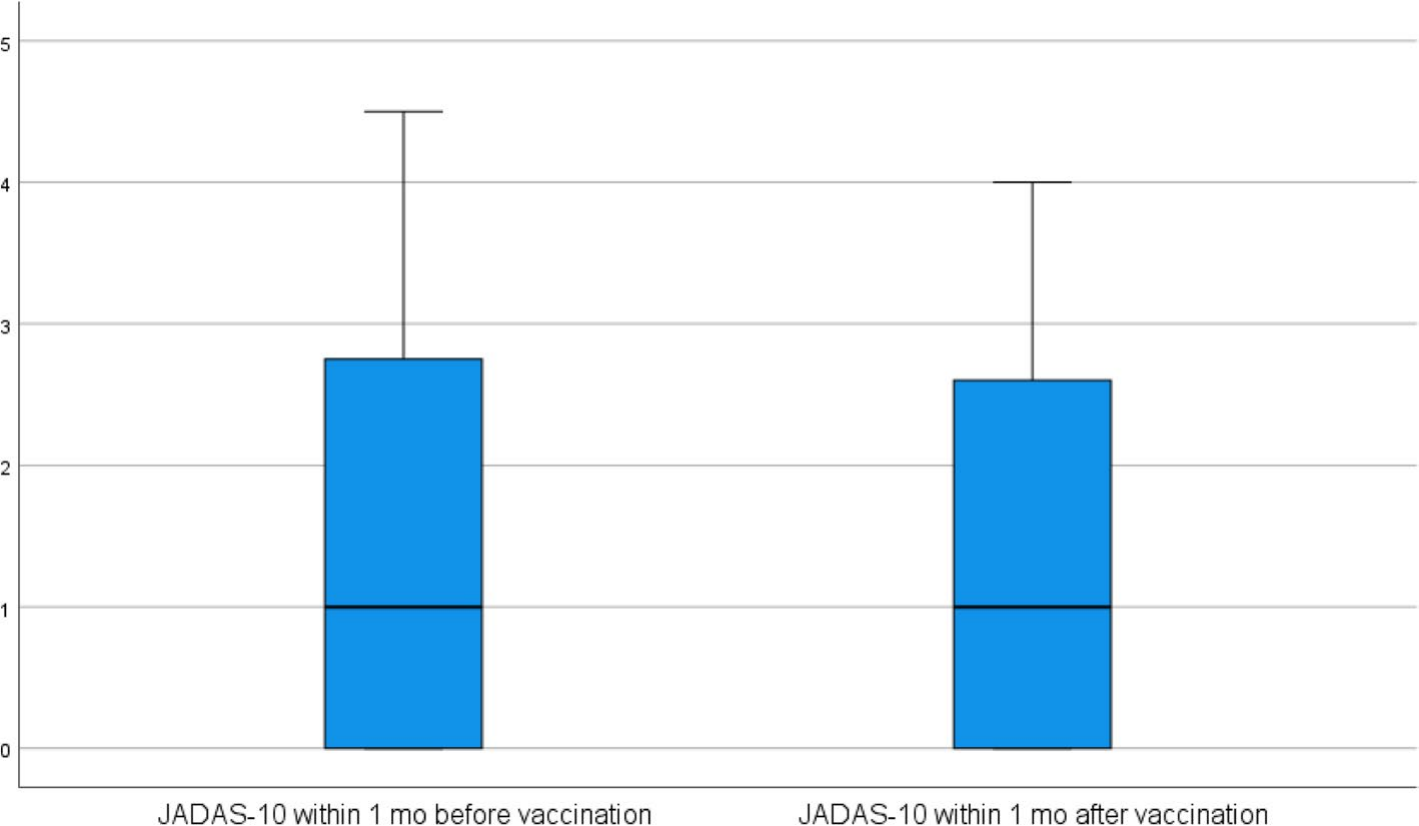
JIA activity by JADAS-10 score before and after vaccination revealing no flare of the primary disease after vaccination

No flaring of the underlying primary disease was reported after vaccination based on nonsignificant changes in scores measured before and after vaccination; JADAS-10; median was one (0.0–4.5) before vs one (0.0–4.0) after vaccination (*p* = 0.9) (Fig. 1). Although there was no statistically significant difference between the frequencies of AE after the 1st and 2nd doses (*p* = 0.5), overall, adverse events were more frequently noted after the 2nd dose (86.1%) rather than after the 1st one (80.6%). Regarding local AE, there was no significant difference regarding age, gender, disease duration, JIA subtypes or disease activity. Turning to systemic AE, post-vaccination nausea was more significantly reported in older patients than younger ones (*p* = 0.03). Moreover, although not statistically significant, fever and headache were also reported in older participants more than younger ones (*p* = 0.07, *p* = 0.08) respectively. There was no significant difference in systemic adverse events among JIA subtypes (Table 3). No one of the study group revealed serious adverse events like anaphylaxis, MIS-C, events that require hospitalization, and also no deaths.

**Table 3:** Correlation between basic characteristics of participants and post-vaccination adverse events

**Post-vaccination adverse events**	**Age** **Mean** (SD)	**Gender** %	**Associated comorbidity** %	**Previous COVID infection** %	**Disease duration** Mean (SD)	**JADAS-10 before vaccination** Mean (SD)
**Local AE (at least one AE)**	Yes 13.8 (3.0) No 12.3 (3.0) *P* = 0.3	F 70.8 M 58.3 P = 0.5	Yes 62.5 No 67.9 *P* = 1	Yes 60 No 67.1 *P* = 1	Yes 4.4 (2.6) No 4.7 (2.7) *P* = 1	Yes 1.3 (1.3) No 1.6 (1.7) *P* = 0.6
Pain at site of injection	Yes 13.6 (3.1) No 12.8 (3.0) *P* = 0.6	F 62.5 M 58.3 *P* = 1	Yes 62.5 No 60.7 *P* = 1	Yes 40 No 64.5 *P* = 0.4	Yes 4.3 (2.7) No 4.9 (2.5) *P* = 0.6	Yes 1.4 (1.4) No 1.5 (1.6) *P* = 0.9
Swelling	Yes 12.7 (3.8) No 13.5 (2.9) *P* = 0.8	F 20.8 M 25 *P* = 1	Yes 37.5 No 17.9 *P* = 0.3	Yes 0 No 25.8 *P* = 0.6	Yes 4.1 (2.6) No 4.6 (2.6) *P* = 0.7	Yes 1.3 (1.1) No 1.5 (1.5) *P* = 0.9
Erythema	Yes 13.8(3.4) No 13.1 (3) *P* = 0.5	F 25 M 25 *P* = 1	Yes 50 No 17.9 *P* = 0.1	Yes 0 No 29 *P* = 0.3	Yes 4.2 (2.6) No 4.6 (2.6) *P* = 0.8	Yes 1.7 (1.3) No 1.3 (1.5) *P* = 0.4
Ipsilateral painful LNs	Yes ( −) ( −) No 13.3 (3.1) *P* = −	F 0 M 0 *P* = −	Yes 0.0 No 0.0 *P* = −	Yes 0 No 0 *P* = −	Yes ( −) ( −) No 4.5 (2.6) *P* = ( −)	Yes ( −) ( −) No 1.4 (1.5) *P* = ( −)
**Systemic AE (at least one AE)**	Yes 13.6 (3.0) No 12.8 (3.2) *P* = 0.4	F 66.7 M 50 *P* = 0.5	Yes 62.5 No 60.7 *P* = 1	Yes 80 No 58 *P* = 0.6	Yes 4.3 (2.7) No 4.8 (2.4) *P* = 0.5	Yes 1.7 (1.5) No 1 (1.3) *P* = 0.2
Fever	Yes 14.2 (1.7) No 12.8 (3.3) *P* = 0.07	F 25 M 25 *P* = 1	Yes 25 No 25 *P* = 1	Yes 20 No 25.8 *P* = 1	Yes 4.4 (2.9) No 4.5 (2.5) *P* = 0.7	Yes 1.9 (1.5) No 1.3 (1.4) *P* = 0.2
Chills	Yes 11.6 (3.2) No 13.5 (3.0) *P* = 0.4	F 4.2 M 25 *P* = 0.1	Yes 12.5 No 10.7 *P* = 1	Yes 0 No 12.9 *P* = 1	Yes 4 (0.8) No 4.6 (2.7) *P* = 0.6	Yes 1.4 (1.1) No 1.4 (1.5) *P* = 0.9
Headache	Yes 14.2 (2.9) No 12.6 (3.1) *P* = 0.1	F 54.2 M 25 *P* = 0.1	Yes 37.5 No 36.4 *P* = 0.7	Yes 80 No 38.7 *P* = 0.1	Yes 4.9 (2.8) No 4.2 (2.4) *P* = 0.4	Yes 1.5 (1.6) No 1.4 (1.4) *P* = 0.7
Diarrhea	*P* = −	*P* = −	*P* = −	*P* = −	*P* = −	*P* = −
Nausea	Yes 15.2 (1) No 12.8 (3.2) ***P* = 0.03**	F 25 M 8.3 *P* = 0.4	Yes 25 No 17.9 *P* = 0.6	Yes 40 No 16.1 *P* = 0.2	Yes 3.9 (1.9) No 4.7 (2.7) *P* = 0.5	Yes 1.5 (1.6) No 1.4 (1.5) *P* = 0.8
Myalgia	Yes 13.3 (2.6) No 13.2 (3.4) *P* = 1	F 37.5 M 50 *P* = 0.5	Yes 25 No 44.4 *P* = 0.4	Yes 80 No 35.5 *P* = 0.1	Yes 3.5 (2.2) No 5.2 (2.3) *P* = 0.06	Yes 1.9 (1.6) No1.1 (1.3) *P* = 0.2
Tiredness	Yes 13.6 (2.8) No 13.1 (3.3) *P* = 0.7	F 45.8 M 33.3 *P* = 0.5	Yes 50 No 58.3 *P* = 0.7	Yes 60 No 38.7 *P* = 0.6	Yes 4.6 (2.9) No 4.5 (2.4) *P* = 0.9	Yes 1.3 (1.2) No 1.5 (1.6) *P* = 1
New transient joint pain	Yes 10.3 (3.1) No 13.6 (3.0) *P* = 0.1	F 4.2 M 16.7 *P* = 0.3	Yes 12.5 No 5.6 *P* = 0.5	Yes 0 No 9.7 *P* = 1	Yes 3 (2) No 4.6 (2.6) *P* = 0.3	Yes 2 (1.0) No 1.4 (1.5) *P* = 0.3
Allergy	Yes 12.5 ( −) No 13.3 (3.1) *P* = 0.7	F 0 M 8.3 *P* = 0.3	Yes 0.0 No11.1 *P* = 1	Yes 0 No 3.2 *P* = 1	Yes 4 ( −) No 4.5 (2.6) *P* = 0.8	Yes 2.5 ( −) No 1.4 (1.5) *P* = 0.6
Flaring of 1ry disease	Yes 7 ( −) No 13.5 (3) *P* = 0.1	F 4.2 M 0 *P* = 1	*P* = −	Yes 0 No 3.2 *P* = 1	Yes 5 ( −) No 4.5 (2.6) *P* = 0.8	Yes 1 ( −) No 1.4 (1.5) *P* = 1
Chest pain	Yes 11.5 (6.4) No 13.4 (2.9) *P* = 0.7	F 8.3 M 0 *P* = 0.5	Yes 0.0 No 8.3 *P* = 1	Yes 0.0 No 6.5 *P* = 1	Yes 5 (0.0) No 4.5 (2.7) *P* = 0.6	Yes 0.5 (0.7) No 1.5 (1.5) *P* = 0.5
Serious AE	*P* = −	*P* = −	*P* = −	*P* = −	*P* = −	*P* = −

*Abbreviations: AE* Adverse events, *JADAS-10* Juvenile Arthritis Disease Activity Score-10

## Discussion

The COVID-19 pandemic is paralyzing the world and, so vaccination is considered of a paramount importance, particularly in children and adolescents with underlying rheumatic disorders with several risk factors, rendering them a vulnerable group, such as immune dysregulation status, use of immunosuppressant medications, and risk of repeated infections [1]. In reviewing the literature, very little was found regarding the safety of the COVID-19 vaccination in the patients with rheumatic disorders. Moreover, comprehensive data regarding COVID-19 vaccination safety in pediatric age groups with underlying rheumatic diseases like JIA is lacking, which has contributed to physician and patient hesitancy and even refusal to get the vaccine [10]. The aim of the current study is to shed new light on the safety of the mRNA vaccine against SARS-CoV-2 (BNT162b2) in children and adolescents with JIA after receiving 2 doses of the vaccine, and also if there is a risk of flaring of the underlying JIA, trying to cross the Rubicon in taking decision of the vaccination which could further prevent infection in this vulnerable group.

In this cohort, the data overall were reassuring. Among the 72 doses of BNT162b2 vaccine received, 66.7% exhibited local adverse events and 65.3% revealed systemic adverse events. Almost all the reported adverse events were mild to moderate and resolved within 1–2 days. In accordance with the current results, Dimopoulou et al. studied safety of the BNT162b2 COVID-19 vaccine in adolescents with JIA on biological therapy and revealed post-vaccination local AE in 74%. However, systemic adverse events were revealed in only 19%, but this study did not include several systemic adverse events like fever, chills, nausea, chest pain and diarrhea [11]. In another study that looked at the safety of another mRNA COVID-19 vaccine on adolescents aged 12–17 years old, systemic adverse events were reported in 68.5% and 86% of the cases after the first and second doses, respectively, and they were generally mild to moderate [12]. Furthermore, almost all the reported adverse events following BNT162b2 vaccination in pediatric populations were mild to moderate and resolved within 1–2 days [13].

Overall, adverse events were more frequently noted after the 2nd dose (86.1%) rather than the 1st one (80.6%) though statistical insignificance (*p* = 0.5). These results match those observed in an earlier study that examined adverse events of BNT162b2 COVID-19 vaccination in JIA adolescents and another study done on normal adolescent populations and revealed a higher incidence of local and systemic adverse events after the second vaccination dose [11, 13]. In contrast to these findings, another study revealed slightly higher post-vaccination adverse events after the first dose (94.2%) than the second one (93.4%). Nevertheless this is a minor difference and, furthermore, the examined vaccine was different (mRNA-1273 SARS-CoV-2) [12].

The most frequently reported local adverse event was pain at the site of vaccine injection (62.5%), consistent with those of previous studies [10, 11, 13]. However, one study revealed a lower percentage (25%) of local pain after injection. A possible explanation for this result may be the reported routine use of acetaminophen in a large number of the study population [14]. Tiredness was the most frequent systemic adverse event (48.6%), followed by myalgia and headache (44.4% and 41.7%) respectively, which agreed with the findings of other studies which studied the safety of different COVID-19 vaccines on adolescents [12, 13, 15]. Nausea was more significantly reported in older patients than younger ones (*p* = 0.03). Moreover, although not statistically significant, fever and headache were also reported in older participants more than younger ones (*p* = 0.07, *p* = 0.08), in agreement with previous studies [13, 16].

Following the vaccination, four patients (five doses (6.9%)) developed mild hives, which were treated with antihistamines and local creams, correlating with previous reports [11]. Similarly, Four patients (five doses (6.9%)); 3 developed mild chest pain which seems to be consistent with other research which found that almost all cases with post-vaccination chest pain were mild and were hemodynamically stable [17].

Reassuringly, no one of the study group revealed serious adverse events like anaphylaxis, MIS-C, events that require hospitalization, and also no deaths, similar to what was described in previous reports that examined possible adverse events of several vaccines against COVID-19 including children and adults populations [11–13, 18, 19]. Hearteningly, no flaring of the underlying primary disease was reported after vaccination based on the scores measured before and after vaccination; JADAS-10 (*p* = 0.9) and also no significant difference in the rate of adverse events among different JIA subtypes that mirror those of the previous research [11]. Furthermore, post-vaccination infection by SARS-CoV-2 was only reported in three patients (8.3%), in agreement with the internationally reported efficacy of the BNT162b2 COVID-19 vaccine 100% (95% CI, 75.3–100) [13].

The main limitations of our study are the small sample size, limited number of patients with JIA subgroups, lack of long-term follow-up of the vaccine safety, lack of control healthy group and also lack of evaluation of the immune response of this novel vaccine in this unique group of JIA patients. So, there will be abundant room for further research trying to clarify these issues.

## Conclusions

In summary, this is one of the early studies reporting that the mRNA COVID-19 vaccine seems to be safe in children and adolescents with JIA. Almost all the reported adverse events were mild to moderate and resolve within 1–2 days. Moreover, no serious adverse events were reported overall. Reassuringly, no flaring of the underlying primary disease was reported after vaccination. This data should be urgently disseminated trying to alleviate vaccine hesitancy in patients with underlying JIA.

## Data Availability

Available upon request.

## Abbreviations

ACR: American college of Rheumatology
AE: Adverse event
COVID-19: Coronavirus disease 2019
DMARD: Disease modifying anti-rheumatic drug
FDA: Food and Drug Administration
ICU: Intensive care unit
JADAS-10: Juvenile Arthritis Disease Activity Score-10
JIA: Juvenile idiopathic arthritis
MIS-C: Multisystem inflammatory syndrome in children
NSAIDs: Non-steroidal anti-inflammatory drugs
PCR: Polymerase chain reaction
SARS-Cov-2: Severe acute respiratory syndrome coronavirus-2
WHO: World Health Organization
